# Cutaneous Memoranda

**Published:** 1886-08

**Authors:** 


3. Cutaneous Memoranda. *By* Henry G. Piffard, a. m., m. d., *Clinical Professor of Dermatology, etc. Third edition. New York:* William Wood & Co. *1885.*

